# Reliability of radioisotope-guided sentinel lymph node biopsy in penile cancer: verification in consideration of the European guidelines

**DOI:** 10.1186/s12894-015-0093-7

**Published:** 2015-09-28

**Authors:** Tim Schubert, Jens Uphoff, Rolf-Peter Henke, Friedhelm Wawroschek, Alexander Winter

**Affiliations:** University Hospital for Urology, Klinikum Oldenburg, School of Medicine and Health Sciences, Carl von Ossietzky University Oldenburg, Rahel-Straus-Straße 10, 26133 Oldenburg, Germany; Oldenburg Institute of Pathology, Taubenstraße 28, 26122 Oldenburg, Germany

**Keywords:** Penile cancer, Sentinel node biopsy, Inguinal lymph node dissection, Lymph node metastases

## Abstract

**Background:**

Lymph node (LN) staging in penile cancer has strong prognostic implications. This contrasts with the high morbidity of extended inguinal LN dissection (LND) or over-treatment of many patients. Therefore, inguinal dynamic sentinel node biopsy (DSNB) or modified LND is recommended by the European Association of Urology (EAU) guidelines to evaluate the nodal status of patients with clinically node-negative penile cancer. This study analyzed the reliability and morbidity of radioguided DSNB in penile cancer under consideration of the current EAU recommendations in an experienced center with long-term follow-up.

**Methods:**

Thirty-four patients who received primary surgery and had radioguided inguinal DSNB for penile cancer (≥T1G2) were included (July 2004 to July 2013). Preoperative sentinel LN (SLN) mapping was performed using lymphoscintigraphy after peritumoral injection of ^99m^Technetium nanocolloid on the day of surgery. During surgery, SLNs were detected using a gamma probe. According to the EAU guidelines, a secondary ipsilateral radical inguinal LND was performed in patients who had positive SLNs. The false-negative and complication rates of DSNB were assessed.

**Results:**

A total of 32 patients were analyzed. Two patients were lost to follow-up. A total of 166 SLNs (median, 5; range, 1–15) were removed and 216 LNs (SLNs + non-SLNs; median, 6; range, 2–19) were dissected. LN metastases were found in five of the 32 (15.6 %) patients and nine of the 166 (5.4 %) SLNs were found to contain metastases. None of the remaining 50 non-SLNs contained metastases. In only one of the five SLN-positive patients, a singular further metastasis was detected by secondary radical inguinal LND. During follow-up (median, 30.5; range, 5–95 months) no inguinal nodal recurrence was detected. DSNB-related complications occurred in 11.1 % of explored groins.

**Discussion and Conclusions:**

Radioguided DSNB is a suitable procedure for LN staging in penile cancer considering the EAU recommendations and with the required experience. Under these circumstances, patients can be spared from higher morbidity without compromising the detection of LN metastases or therapeutic implications. Improvement of the methodology used to perform DSNB should be developed further to decrease the risk of missing LN metastases and to simplify the procedure.

## Background

In penile cancer, lymph node (LN) metastasis is the main known prognostic factor affecting patients’ survival [1]. A recent published analysis showed an overall 5-year cancer-specific survival of patients with primary invasive tumors of 81 %, but only 56 % of patients with LN metastases survived the first 5 years after diagnosis [2]. Inguinal LNs are the first nodal group affected in penile cancer. Early inguinal LN dissection (LND) or the resection of clinically occult LN metastases improves survival compared with removal when the metastases become clinically apparent [2–4]. Only one-third of penile cancer patients with regional recurrence are alive after 5 years [4].

Consequently, management of inguinal LN is crucial for prognosis in patients with penile cancer. Patients with palpable inguinal nodes are at high risk for LN metastases. In patients with nonpalpable inguinal nodes (cN0), the likelihood of the presence of metastasis is approximately 20–25 % [5]. However, current imaging techniques are not reliable for detecting micro-metastases [6]. Moreover, nomograms are also inappropriate for predicting LN metastases in penile cancer. The accuracy of currently available predictive models is below 80 % [6].

Therefore, in cases of palpable inguinal LNs, radical inguinal LND is required. Patients with normal inguinal nodes and an intermediate or high risk of lymphatic metastasis (≥T1G2) should also receive invasive LN staging [6]. Because only 20–25 % of patients with normal inguinal LNs experience regional lymphatic spread, performing a radical inguinal LND may be an overtreatment in approximately 80 % of these cases, resulting in considerable morbidity [7]. Complications, such as wound infection, skin necrosis, wound dehiscence, lymphedema, and lymphoceles, can occur [8].

To reduce the morbidity associated with radical inguinal LND, two invasive procedures are recommended by the European Association of Urology (EAU) guidelines for patients with nonpalpable nodes: modified inguinal LND and dynamic sentinel node biopsy (DSNB) [6]. If either modified LND or DSNB show LN metastases, an ipsilateral radical inguinal LND should be performed. Fine needle aspiration cytology (FNAC) has been advised in clinically node-negative patients by former guidelines [9], but it is no longer recommended by the EAU owing to low specificity [6]. Moreover, FNAC is not reliable for the detection of micro-metastases.

Modified inguinal LND includes a limitation of the dissection field and preservation of the saphenous vein. As a result, morbidity of the inguinal LND procedure can be reduced. However, limitation of the dissection area results in a higher probability of false-negative cases [8]. According to the current EAU guidelines, the false-negative rate of modified inguinal LND is not known [6]. There are few data on this aspect of this disease.

DSNB in penile cancer was first described by Cabanas [10]. The modern sentinel concept in penile cancer using a radioactive tracer with or without blue dye was introduced around the turn of the millennium and was recently further developed [5, 11, 12]. The reliability and low morbidity of this technique have been reported by various research groups [7, 13–16].

This study aimed to analyze the reliability of radioisotope-guided DSNB in penile cancer in an experienced center under consideration of the current EAU recommendations and with a long-term follow-up.

## Methods

### Patient population and inclusion criteria for analysis

A total of 49 patients with penile cancer were operated on at our institute from July 2004 to July 2013 and documented in a consecutive data bank. In this retrospective study, 34 patients with ≥ T1G2 tumors who received radioisotope-guided inguinal DSNB were included. Of these, 32 could be analyzed. Two patients died a few months after surgery. Causes of death were not related to complications of the operation or penile cancer.

All of the patients were informed orally and in writing regarding inguinal DSNB and penile surgery, and they gave informed consent.

### Surgical treatment

The DSNB was performed in accordance with the current EAU guidelines [6]. A FNAC, which is still recommended by others to reduce the false-negative rate, was not performed [9].

Surgical treatment of the primary tumor depended on the tumor stage, and included circumcision (*n* = 5), resection of the glans with or without circumcision (*n* = 11), or partial resection of the penis (*n* = 16). All included patients presented with a tumor stage ≥ T1G2 and received an inguinal DSNB. In 17 patients, the DSNB was performed in a one-step manner in the same operation. Fifteen cases received a secondary inguinal DSNB. Two patients additionally received stage-adapted modified or radical inguinal LND. In one of these patients, radical LND of the left groin and a DSNB of the right groin were performed. The radical LND was indicated because of an ipsilateral suspicious LN. The other patient had bilateral enlarged inguinal LNs.

According to the EAU guidelines, a secondary ipsilateral radical inguinal LND was performed in patients who had positive SLNs (pN1 stage). Secondary inguinal and pelvic LND was performed if histopathological evaluation of the inguinal LND specimen revealed two or more tumor-positive LNs (pN2 stage).

### Sentinel tracer injection

**Fig. 1:**
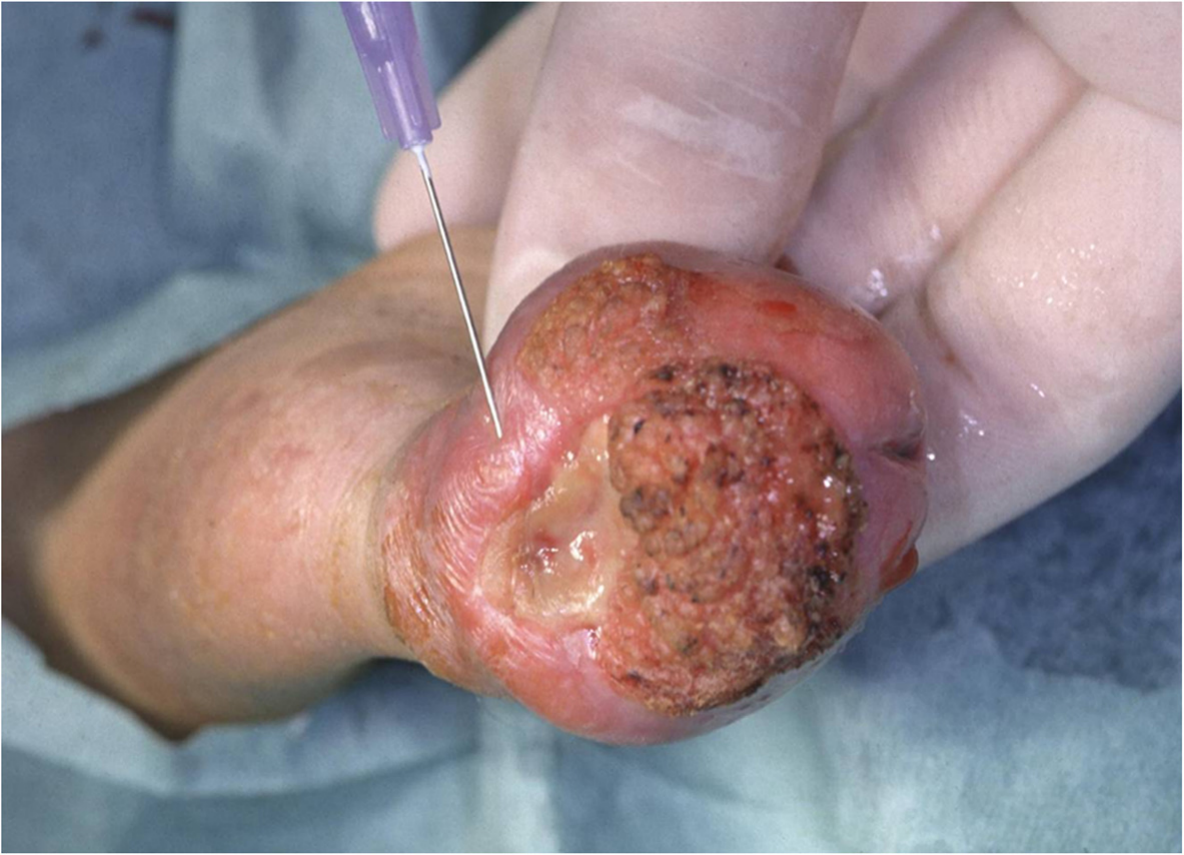
Peritumoral injection of the sentinel tracer (^99m^Technetium nanocolloid)

The sentinel tracer (^99m^Technetium nanocolloid, radioactivity ca. 30 MBq) was preoperatively injected peritumorally on the day of surgery approximately 4 hours before the operation (*n* = 17) (Fig. 1) or in a two-step procedure in the area of the resection (*n* = 15). Lymphoscintigraphy was then carried out. The SLNs were intraoperatively detected using two different gamma probes (C-Trak System, Care Wise, Morgan Hill, CA, USA; Crystal Probe SG04, Crystal Photonics GmbH, Berlin, Germany).

### Intraoperative procedures and histopathology

LNs identified as SLNs by the gamma probe were dissected. For surgical reasons, LNs other than SLNs directly adjoining and adhering to SLNs were also removed, if in situ separation was not possible.

All LNs were initially cut in 3-mm transverse sections, routinely processed, and completely embedded in paraffin. Sections that were 4–5 μm thick were stained with hematoxylin–eosin.

### Follow-up

All intra- and postoperative complications were recorded according to the Clavien–Dindo classification. Follow-up was performed on an outpatient basis by urologists in the community in accordance with our instructions. As recommended by the EAU guidelines [6], for patients with negative inguinal nodes after local treatment, follow-up visits included physical examination of the penis and the groin for detection of local and/or regional recurrence. On indication, ultrasound, computed tomography (CT), or magnetic resonance imaging (MRI) was used. In patients with positive LNs, CT or MRI scanning was carried out at 3-monthly intervals during the first 2 years for the detection of regional recurrence or systematic disease.

### Analysis

We classified DSNB as a false-negative procedure only if non-SLNs were positive or regional nodal recurrence developed after a negative SLN procedure. The false-negative rate of DSNB was calculated according to the standard definition: false-negative procedures/true-positive procedures + false-negative procedures.

### Ethical approval

The study was approved by the Ethics Committee of the Carl von Ossietzky University Oldenburg.

## Results

A summary of the patient and tumor characteristics is shown in Table 1.

**Table 1:** Summary of patient and tumor characteristics

Characteristic	No. of patients
Total	32
Grade by T stage	
T1	20
G1	excluded from study
G2	18
G3	2
T2	10
G1	1
G2	9
G3	0
T3	2
G1	0
G2	0
G3	2
T4	0
Follow-up, month	
Median	30.5
Range	5–95
Age, years	
Median	67
Range	39–78

A total of 166 SLNs (median, 5; range, 1–15) were removed and 216 LNs (SLNs + non-SLNs; median, 6; range, 2–19) were dissected. LN metastases were found in five of the 32 (15.6 %) patients and nine of 166 (5.4 %) SLNs were found to contain metastases. None of the remaining 50 non-SLNs contained metastasis. Of the five patients with positive SLNs, three presented with pN1 stage. In secondary radical inguinal LND, a singular further metastasis was detected only in one of these patients, while the other two patients had pN2 stage disease. These two patients were also clinically node positive. One of these patients, who showed preoperative enlarged LNs bilaterally, was the only patient with LN metastases in both groins. All other nodal positive patients presented with unilateral metastases. On secondary iliacal LND in one of the two cases, pelvic LN metastases were found (3 of 11 removed LNs were positive). In the other patient, all further LNs were free of metastases. The histopathological findings are shown in detail in Table 2. During follow-up, no inguinal nodal recurrence was detected. Accordingly, no patient had false-negative DSNB taking into account the EAU recommended procedure, including a secondary radical LN in the case of positive SLNs.

**Table 2:** Nodal status related to histopathological tumor stage

Stage	pN0	pN+ inguinal	pN+ pelvic
	(*n* = 26)	(*n* = 5)	(*n* = 1)
pT1 G2	16 (89 %)	2	0
pT1 G3	1 (50 %)	1	1
pT2 G1	1 (100 %)	0	0
pT2 G2	8 (89 %)	1	0
pT3 G3	1 (50 %)	1	0

Recurrence of primary tumors was identified in two patients at 11 or 12 months after surgery by physical examination or by MRI. In one patient, a Merkel cell carcinoma of the right thigh was diagnosed simultaneously with penile cancer. During follow-up, diffuse metastasis of the Merkel cell carcinoma appeared.

Four patients died during the follow-up. The median follow-up for these patients was 44.5 months (range, 22–69 months). Reasons for death were pulmonary emphysema, advanced renal cell carcinoma, and systemic metastatic disease. In one patient, the cause of death remained unclear.

DSNB-related complications were assessed in a total of five patients, but intervention was only required in three patients (Clavien–Dindo grade III). One patient underwent a revision operation of both groins owing to wound infection. Wound healing was achieved by vacuum bandages and antibiotics after the revision operation (Clavien–Dindo grade IIIb). Two patients had unilateral inguinal lymphoceles. In these two patients, the lymphoceles were drained (Clavien–Dindo grade IIIa). Two other patients suffered from prolonged wound secretion of the groin and were treated with clinical surveillance without any re-intervention procedures (Clavien–Dindo grade I). Five patients had complications (Clavien–Dindo grade III) after radical LND of the groins. Two patients underwent a revision operation of one groin owing to wound infection (Clavien–Dindo grade IIIb). Wound healing was achieved either by vacuum bandages and antibiotics after the revision operation or secondary operation. Drainage of lymphoceles was performed in the other three patients (Clavien–Dindo grade IIIa). Therefore, the complication rate concerning DSNB alone was 11.1 % (per groin). Complications occurred in all patients with radical inguinal LND (100 %, per groin). In these patients, an intervention was always required.

## Discussion

In this study, we performed a retrospective analysis of patients who underwent radioisotope-guided DSNB in a center with wide expertise in sentinel procedures related to urological malignancies. The reliability of this approach was investigated under consideration of the current EAU guidelines (2014) [6]. The median follow-up was 30.5 months in the present study. Because recurrence of tumors occurs typically within 2 years, a false-negative rate of DSNB should become clinically apparent at that time [17]. Accordingly, a median follow-up of 30.5 months is sufficient to address this issue. This is underlined by the fact that, in our study, no inguinal recurrence was detected if patients who were only followed up for at least 24 months (*n* = 19; median follow-up, 60 months; range, 24–95 months) were analyzed.

A DSNB or an EAU recommended procedure [6], including secondary LND in patients with positive SLNs, showed reliable results in the examined population. In only one patient with a tumor-positive SLN, a singular further LN metastasis was detected on secondary radical LND. None of the patients suffered recurrence of inguinal LN. Our results are in line with recent studies in which DSNBs showed high reliability. In a prospective study, Lam et al. analyzed a total of 264 patients with penile cancer undergoing DSNB [14]. The false-negative rate per patient was 6 %. Fuchs et al. found an inguinal nodal recurrence in only 3.7 % of their patients [18], while Leijte et al. investigated 323 patients and calculated a false-negative rate of 7 % per groin [19]. In a recent national multicenter study from Denmark, the overall false-negative rate was 13.3 % per patient [20]. However, caution is advised, because in our study, 56 % of the patients had a low stage (T1G2). At a median of 30 months, there are still many patients at risk for recurrence. For this reason, our results may not reflect the outcome of series with a different mixture of patients. In an initial DSNB series by Kirrander et al., the recurrence rate was 15 % [21]. However, in their study, 50 % of the patients had T2 or greater primary tumors.

Some more potential limitations of this study need to be discussed. The results from the present study are based on the data of one center and on a relatively small cohort. In this context, the low incidence of penile cancer in Germany has to be taken into account. However, this study represents the largest published German DSNB series in penile cancer to date.

Another limitation is the fact that the DSNB was performed either primarily or secondarily after resection of penile tumors in the present study. LN metastases were found in five patients. Three patients underwent primary DSNB, and two underwent a secondary procedure after resection of the primary tumor. However, a study by Graafland et al. suggested that the results of a primary DSNB were equal to those of a two-step procedure [22]. In their study, 40 patients who had undergone DSNB after previous resection of the primary penile tumor were analyzed, and no recurrences developed in the groins during a median follow-up of 28 months.

Whether a DSNB should be performed is still controversial, mostly because a false-negative result is associated with a significant risk of death. Therefore, some authors have stressed that a DSNB cannot be considered as a real gold standard for evaluation of patients with cN0 penile carcinoma [23, 24]. This is because initial results of DSNB in penile cancer show a high false-negative rate of 19.2–22 % [7, 25]. In a recent study, an experienced research group in Amsterdam showed that the 5-year cancer-specific survival for all patients with pN+ disease is better than that in series that prefer primary inguinal LND in all patients who are considered at risk for LN metastases [2]. After performing several modifications to the DSNB procedure, the false-negative rate dropped to 4.8 % [7]. As mentioned above, other studies have shown that DSNB is a reliable and safe method for inguinal LN staging in penile cancer [7, 13–16]. Moreover, excellent results concerning sensitivity, false-negative rates, and complication rates for DSNB have been reported in several studies [14, 19, 26]. These findings show the importance of methodology in DSNB. However, the conclusion cannot be made that a lower false-negative rate is only caused by these modifications; experience in performing DSNB might also be a contributing factor.

Re-routing of the radioactive tracer due to tumor blockage of lymph vessels has been proposed as a mechanism for increasing the risk for false-negative SLN procedures [27]. One fundamental problem with the sentinel technique is that when LNs are fully metastasized or lymph pathways are blocked, the afferent lymph will be directed to other LNs/non-SLNs [28]. Therefore, Leijte et al. added preoperative ultrasound of the groins with FNAC of suspicious nodes to identify and cytologically examine possible blocked SLNs [7]. This procedure was advised in clinically node-negative patients in former guidelines [9], but it is no longer recommended by the EAU owing to the low specificity of FNAC [6]. The current EAU guidelines take the possibility of fully metastasized LNs or blocked lymphatic vessels into account by recommending a primary radical inguinal LND in patients with clinical enlarged LNs or a secondary radical LND in cases with tumor-positive SLNs, respectively.

Further investigations have been made to improve the reliability of DSNBs. Brouwer et al. recently showed indocyanine green-^99m^Technetium-nanocolloid as a hybrid radioactive and fluorescent tracer for performing DSNB [12]. This tracer was successfully used for combined radio- and fluorescence-guided DSNB in penile carcinoma. A large improvement in optical SLN detection compared with blue dye has been achieved using this new approach.

The low risk of a false-negative rate in DSNB has to be weighed against the morbidity of conventional inguinal LND in penile cancer. Inguinal LND is a potential over-treatment in patients without regional LN involvement, which constitutes about 80 % of those with cN0 disease [29]. The rate of complications of DSNB in the present study was 11.1 % (per groin), which is in line with recent studies [21, 30]. We found no cancer in non-SLNs. This suggests that more extensive node sampling is unnecessary and may contribute to morbidity. Other authors have reported a lower rate of complications after DSNB [7, 14, 18, 24]. Leijte et al. were able to decrease the complication rate of inguinal DSNB from 10.2 to 5.7 % [7]. In a systemic review, Neto et al. reported a complication rate of 3.6 % when performing inguinal DSNB [31]. However, inguinal LND is a procedure with a considerably higher complication rate of 40–50 % [23, 31]. Protzel et al. pointed out that the potential advantage of reduced morbidity with DSNB appears to be less pronounced compared with modified inguinal LND [8]. However, there are only limited data regarding the false-negative rate of modified LND [6]. Therefore, further studies are needed to compare the false-negative rates of DSNB and modified LND.

## Conclusions

Radioguided DSNB is a suitable procedure for LN staging in penile cancer patients under consideration of the EAU recommended procedure in experienced centers. Under these circumstances, patients can be spared from higher morbidity without compromising the detection of LN metastases or therapeutic implications in LN positive patients. Improvement of the methodology used to perform DSNB (e.g., new tracers) should be developed further to decrease the risk of missing LN metastases and to simplify the procedure.

## Abbreviations

CT: Computed tomography
DSNB: Dynamic sentinel node biopsy
EAU: European Association of Urology
FNAC: Fine needle aspiration cytology
LN: Lymph node
LND: Lymph node dissection
MRI: Magnetic resonance imaging
SLN: Sentinel lymph node
